# Photoacoustics of single laser-trapped nanodroplets for the direct observation of nanofocusing in aerosol photokinetics

**DOI:** 10.1038/ncomms10941

**Published:** 2016-03-16

**Authors:** Johannes W. Cremer, Klemens M. Thaler, Christoph Haisch, Ruth Signorell

**Affiliations:** 1 Department of Chemistry and Applied Biosciences, Laboratory of Physical Chemistry, ETH Zurich, Vladimir-Prelog-Weg 2, CH-8093 Zurich, Switzerland; 2 Laboratory for Applied Laser Spectroscopy, Chair of Analytical Chemistry, Technical University of Munich, Marchioninistrasse 17, D-81377 Munich, Germany

## Abstract

Photochemistry taking place in atmospheric aerosol droplets has a significant impact on the Earth's climate. Nanofocusing of electromagnetic radiation inside aerosols plays a crucial role in their absorption behaviour, since the radiation flux inside the droplet strongly affects the activation rate of photochemically active species. However, size-dependent nanofocusing effects in the photokinetics of small aerosols have escaped direct observation due to the inability to measure absorption signatures from single droplets. Here we show that photoacoustic measurements on optically trapped single nanodroplets provide a direct, broadly applicable method to measure absorption with attolitre sensitivity. We demonstrate for a model aerosol that the photolysis is accelerated by an order of magnitude in the sub-micron to micron size range, compared with larger droplets. The versatility of our technique promises broad applicability to absorption studies of aerosol particles, such as atmospheric aerosols where quantitative photokinetic data are critical for climate predictions.

## Abstract (web summary)

Aerosol droplets have significant effects on atmospheric photochemistry, however measuring absorption from single droplets is a challenge. Here, the authors report a method to measure absorption of nanodroplets with attolitre sensitivity, showing rate enhancements for light focusing in photolysis reactions.

## Graphical abstract

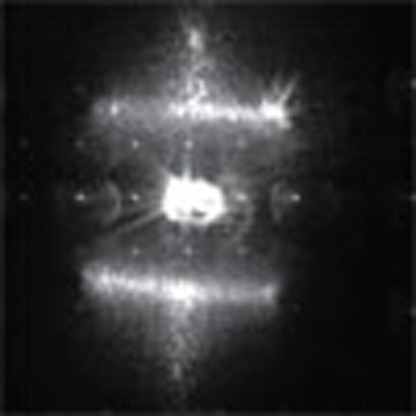

Understanding fundamental processes that govern the reaction dynamics of gas phase, aerosol and cloud processes is crucial for the advancement of global atmospheric chemistry modelling^1^,^2^,^3^,^4^,^5^,^6^,^7^,^8^,^9^,^10^,^11^,^12^,^13^,^14^. Much of the chemistry occurring in the Earth's atmosphere is driven by sunlight. Photochemical reactions, in which aerosol particles or droplets act as the active reaction medium, can be highly complex because they are influenced by optical phenomena, transport properties and surface effects^2^. Optical phenomena play a fundamental role in light-initiated particle processes since the radiation flux within the particles determines the activation rate of the photochemically active species.

Focusing of electromagnetic radiation inside small particles leads to an enhancement of the overall light intensity, compared with the intensity of the incident radiation and to structuring and localization of the internal optical fields^15^,^16^,^17^,^18^,^19^,^20^,^21^,^22^,^23^. These phenomena depend strongly on the particle size, the particle composition and the wavelength of electromagnetic radiation. The fundamental influence of the enhanced electromagnetic energy density on the rate of photochemical reactions in micro- and nanodroplets has been recognized and calculations have provided limited evidence for enhanced photochemical rates^24^,^25^,^26^,^27^. Experimental results remain inconclusive concerning the influence of light enhancement on the kinetics, mainly because direct observation of the actual photoactive step was not possible^23^,^28^,^29^,^30^,^31^,^32^,^33^,^34^. The observation of size-dependent effects in ensembles of aerosol or emulsion droplets is often hindered because the droplet size distribution cannot be varied and determined with the necessary accuracy. However, even single-droplet techniques have so far not provided size-dependent photolysis rates because the direct measurement of the population decay of the photoactive substance was not possible.

Elastic light scattering is sensitive enough to allow measurements on single sub-micron-sized droplets, but the information content is not specific enough to extract size-dependent rates. Raman spectroscopy, by contrast, could provide specific information but it comes with the disadvantage of low sensitivity (long averaging times), which would make its application to study processes in single submicrometre droplets where nanofocusing becomes important very challenging. Single-droplet fluorescence studies require a fluorescing compound, which strongly restricts its applicability. Furthermore, the fluorescence depletion is not always a reliable measure of the population decay of the photoactive species because of varying quenching efficiencies. The recently presented cavity ring-down studies on single droplets provide information on the extinction but not directly on the droplet absorption^35^,^36^. Even in combination with light scattering measurements, the determination of rates in nanodroplets is likely prohibited by the uncertainty of the derived absorption.

This study reports the direct observation of light nanofocusing on the photokinetics in nanometre- to micron-sized droplets in the ultraviolet/visible (UV/VIS) range. To this end, we introduce single-droplet photoacoustic (PA) absorption spectroscopy, allowing the direct detection of the population decay of the photoactive substance. PA spectroscopy has been successfully used for the investigation of ensembles of aerosol particles^37^,^38^,^39^,^40^, but its applicability to single aerosol particle studies has been controversial and has not previously been realised experimentally. Here we demonstrate the feasibility of single-droplet PA spectroscopy in combination with laser trapping, and provide direct experimental evidence for the size-dependence of the photolysis rate in model aerosol droplets due to nanofocusing effects. The results are compared with simulations using classical cavity electrodynamics.

## Results

### Principle of single-droplet PAs

**Figure 1:**
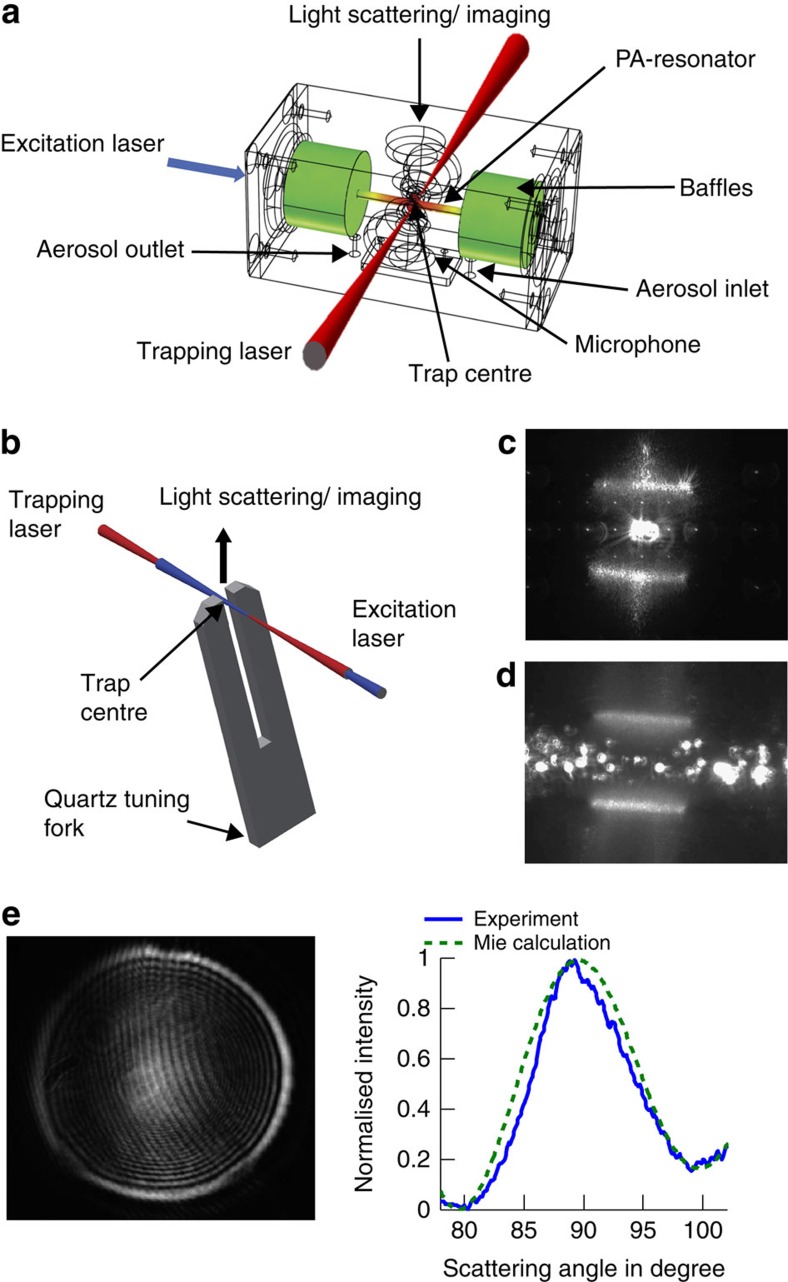
Sketch of the experimental single-droplet PA set-ups. (**a**) Microphone set-up with PA-resonator, excitation laser, trapping laser and light scattering measurements. The colours in the PA cell indicate that the acoustic mode has its maximum amplitude (red) in the vicinity of the microphone and a value close to zero (green) in the region of the acoustical baffles. (**b**) Tuning fork set-up. (**c**) Snapshot of a single droplet trapped between the tines of the tuning fork (view from top). Note that the droplet (∼1 μm) is much smaller than the detection volume between the tines (∼0.3 × 0.34 × 2 mm). (**d**) Snapshot of an ensemble of droplets flowing in between the tines from left to right. (**e**) Light scattering image as recorded by the CMOS camera (left) with experimental and calculated phase function (right) for a droplet with a radius of *a*=530 nm.

The two experimental set-ups, using a microphone and a quartz tuning fork, respectively, for resonant single-droplet PA measurements, are sketched in Fig. 1a,b (see Methods). For the droplet absorption experiments, we use a *λ*=445 nm excitation laser (Nichia laser diode NDB7112E) of variable power (0.3–40 mW) modulated at the resonance frequency of the PA-resonator and the tuning fork, respectively. The resonance frequency and the *Q*-factor of the PA-resonator and the tuning fork are 3.97 kHz and ∼8.9, and 32.7 kHz and ∼8,000, respectively (Supplementary Fig. 1). The power of the excitation laser is recorded by a power meter after passing the PA cell and the tuning fork, respectively (Supplementary Fig. 2). The amplified PA signals are averaged over either 30 ms or 200 ms. For single-particle trapping, we use a counter-propagating optical tweezer built from a continuous laser beam of *λ*=660 nm of ∼200 mW (Laser Quantum, Opus 660) (Supplementary Fig. 2). Such multiple beam optical traps allow trapping of sub-micron droplets, and combine the advantage of a comparatively simple set-up with high trapping stability and tight particle confinement (<100 nm)^41^,^42^,^43^. Droplets are trapped by gradient forces pointing towards the trap centre for all translational degrees of freedom. Single droplets are captured in the trap centre from a plume of aerosol generated by a nebulizer (see Methods). The droplet size is determined from laser light elastically scattered by the droplet^41^,^44^ (see Methods, Supplementary Fig. 3). Figure 1e shows an example for light scattering measurements for droplet sizing. In the microphone set-up (Fig. 1a), the trap centre is located in the middle of the PA-resonator above the microphone^45^. The trapping and excitation lasers enter and exit the cell through wide-band, anti-reflective windows coated for the respective wavelengths. The CMOS camera for particle imaging and light scattering measurements is placed perpendicular to the excitation and trapping laser. The aerosol inlet and outlet are on the side of the PA cell outside the resonator.

In the tuning fork set-up (Fig. 1b), the droplet is trapped between the tines of the fork with collinearly aligned excitation laser and trapping laser beams. The CMOS camera for particle imaging and light scattering measurements is placed opposite the tines of the fork. Figure 1c,d shows images of a single droplet trapped in between the tines and of a droplet ensemble flowing through the tines, respectively. The principal attractiveness of the tuning fork derives from its high detection sensitivity (very high *Q*-factors) and low sensitivity to environmental acoustic noise^46^. In our set-up, we mainly profit from the ease of combining it with laser trapping and light scattering measurements, as well as from the fact that it is chemically inert.

### PA response of a single droplet

**Figure 2:**
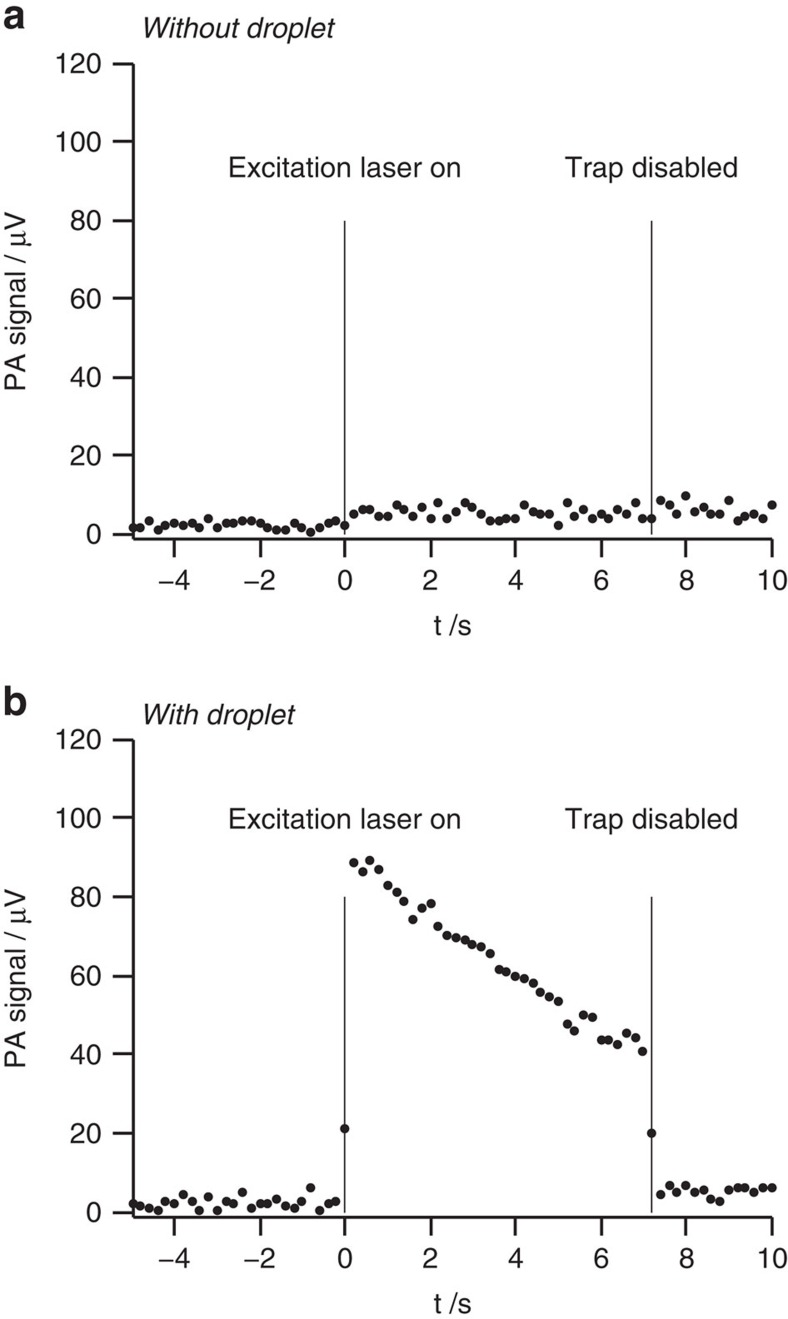
Typical noise levels and background signals for the microphone set-up. (**a**) For the empty trap, identical noise levels and background signals are recorded for a pure TEG solvent droplet in the trap (data not shown). (**b**) With a VIS441/TEG solution droplet in the trap. At *t*=0 s, the excitation laser (445 nm) is switched on and at *t*=7 s the trapping laser is switched off.

Figure 2 provides typical noise levels, background signals and a proof for single-droplet detection. Figure 2a illustrates the noise level and the background signal for the empty trap with the trapping laser on. With the excitation laser off (−5 s<*t*<0 s), the background signal (average) and noise level (1 s.d.) are ∼2.2±1.2 μV. Once the excitation laser is turned on at time *t*=0 s, a background signal of *BS*=5.3 μV with a noise level of *NL*=1.7 μV is recorded. The background signal is caused by excitation laser light scattered from the cell walls and hitting the microphone. Blocking the trapping laser (that is, disabling the trap) leaves the noise, as well as the background unchanged.

**Figure 3:**
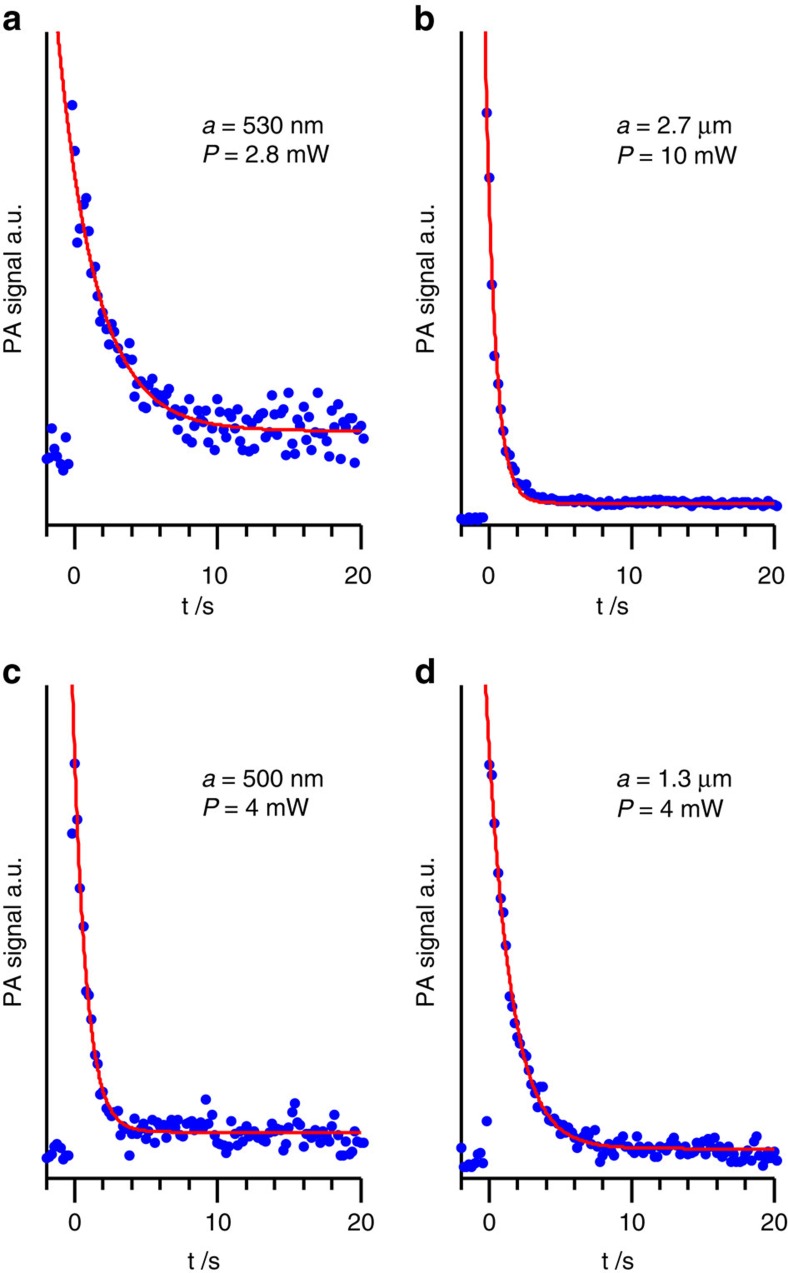
Exemplary PA signals of VIS441/TEG solution droplets as a function of time. The decay of the signal is caused by photolysis of the solute. The experimental data (blue dots) are recorded for different droplet radii *a* and different power *P* of the excitation laser. (**a**,**b**) Recorded with the microphone set-up. (**c**,**d**) Recorded with the tuning fork setup. The red lines are fits to the experimental data providing experimental first half-lives *t*_1/2_ (see Fig. 4).

Figure 2b shows the same as Fig. 2a but with a single VIS441/tetraethylene glycol (TEG) solution droplet in the trap. The PA signal reaches a maximum (*S*_max_) just after the excitation laser is turned on and then decreases exponentially as the VIS441 absorber undergoes photolysis. In Fig. 2b, the trap is disabled at *t*=7 s which leads to the immediate loss of the droplet and to a decrease of the PA signal to the background signal BS. This proves that the signal between 0 s<*t*<7 s indeed comes from a single droplet. The signal to noise ratio 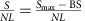 depends on the power *P* of the excitation laser, the concentration of the solution and the droplet size. Figure 3 shows exemplary experimental data for solution droplets of different size excited with different laser powers *P*. With the tuning fork set-up, we find an improvement in the 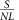 ratio of a factor of ∼3 compared with the microphone set-up. Note that the PA signal is caused by absorptive heating of the droplet and subsequent heat transfer to the surroundings. Evaporation of the solvent does not occur.

### Detection limit

The minimum absorbance detectable with the single-particle PA set-ups at a given power of the excitation laser can be estimated from the PA signal and the single-droplet absorbance assuming that the noise level *NL* is the detection limit (see Methods, Calculation of droplet absorbance). As an example, we use the PA signal at *t*=0 (*S*_max_) of the 530 nm droplet shown in Fig. 3a recorded at a laser power of 2.8 mW with an averaging time of 200 ms. The refractive index of this droplet, *n+ik*=1.463*+i*·0.0062 (Methods and Supplementary Figs 4 and 5), yields an absorption cross-section of *C*_abs_=0.22 μm^2^. (*S*_max_−*BS*)=33.3 μV corresponds to an equivalent absorbance of *A*=1.8 × 10^−5^. From the measured *NL* of the 530 nm droplet of *NL*=1.7 μV, we derive a minimum absorbance *A*_min_=9 × 10^−7^ detectable with the microphone set-up. The improvement in 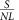 by a factor of ∼3 for the tuning fork set-up reduces the detection limit to *A*_min_∼3 × 10^−7^, a minimum detectable absorption coefficient of *α*_min_=0.0074 × 10^−6^ m^−1^ or a minimum detectable absorption cross-section *C*_abs,min_=0.0037 μm^2^ (laser power of 2.8 mW and averaging time of 200 ms) (see Methods equations (2)–(4), 10, 11). The equivalent particle radius of 146 nm corresponds to a probe volume of 13 al. This far exceeds the performance of typical spectrometers (*A*_min_∼10^−3^–10^−4^), and is at least comparable to the most sensitive laser spectroscopic absorption measurements for macroscopic probe volumes. Note that in our set-up, this sensitivity is achieved for small (attolitre) probe volumes and very short (<<1 s) measurement times. Both can be further improved by increasing the laser power.

### Size-dependent photokinetics

The photokinetics in small droplets do not follow simple pseudo first order kinetics because the light intensity distribution inside the droplets is time dependent; that is, because of the concentration dependence of the nanofocusing. Therefore, we use the same initial concentration for all experiments so that the first half-life can be used as a measure for the size-dependence of the photokinetics. With our PA set-up, we directly measure the decay in absorption resulting from the population decay of the photoactive substance. Diffusion is so fast in the droplets (∼10 μm^2^ s^−1^) that concentration gradients cannot build up and homogeneous concentrations for the solute can be assumed at all times. A model for the droplet photokinetics under these conditions is provided in Methods (Calculation of droplet photokinetics).

**Figure 4:**
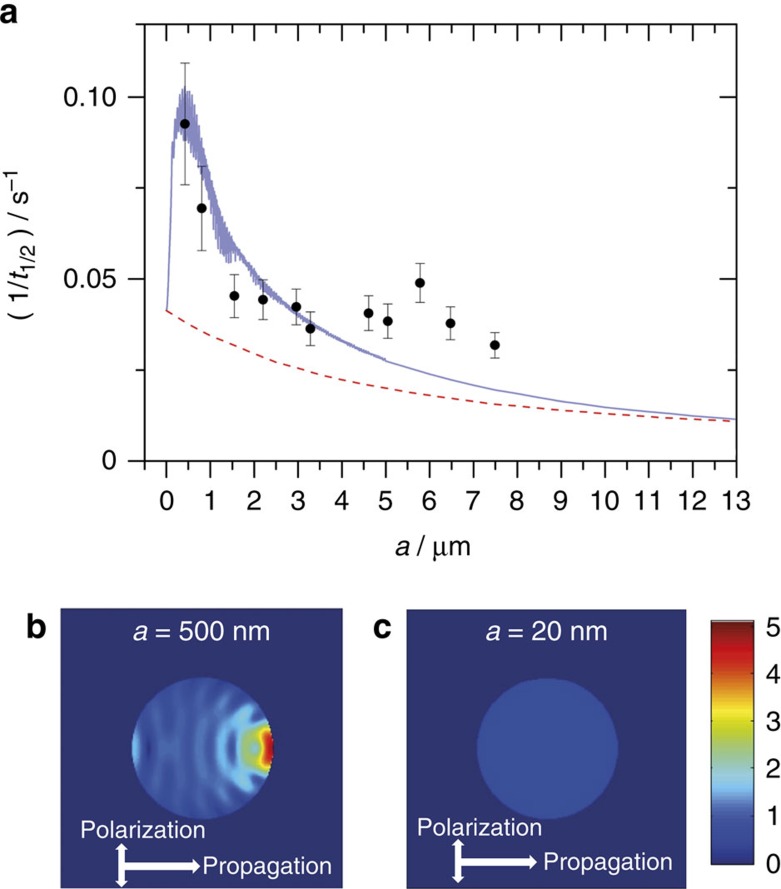
Size-dependent photokinetics. (**a**) Inverse first half-lives 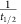 as a function of the droplet radius for a laser power of 1 mW. Black circles: statistically evaluated experimental data. Error bars show 1 s.d. Full blue line: model prediction including nanofocusing and scattering effects. The calculations are for a quantum yield of 7 × 10^−6^. Dashed red line: model prediction for a hypothetical bulk limit, that is, excluding contributions from nanofocusing and scattering. Distribution of the light intensity inside droplets at *t*=0 s for (**b**) a 0.5 μm droplet and (**c**) a 20 nm droplet. The colour scheme is relative to an incident light intensity of 1.

The model prediction (full blue line in Fig. 4a) shows a strong droplet size-dependence with a maximum in the inverse first half-life 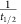 at a droplet radius of ∼0.5 μm. Pronounced increases in 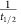 and fluctuations due to resonances are observed over the droplet size range from ∼50 nm to ∼1.2 μm. In this size range, the increase of the rate is caused by the enhancement of the internal electromagnetic field intensity through focusing of the light inside the droplet. Figure 4b shows the distribution of the light intensity inside a ∼0.5 μm droplet at *t*=0 s. The enhancement of the overall intensity and the local variation of the intensity are pronounced. The inverse half-life at a droplet radius of ∼0.5 μm is increased by a factor of ∼2.5 compared with the infinitely small droplet limit. The kinetics in these small droplets is no longer influenced by nanofocusing inside the droplet or light scattering by the droplet as visualized by the internal field intensity in Fig. 4c. The inverse half-life of larger droplets (>6 μm) exhibits only a weak size-dependence but decreases continuously (towards zero for infinitely large particles). The rate for these large droplets is determined by the balance between the decay of the absorber and the rise of the decay rate with time. As the photolysis proceeds, the penetration depth of the light and hence the internal field intensity increases. As in the case of very small droplets, nanofocusing is not important for very large droplets. Large droplets essentially represent the behaviour of thin bulk films with the same effective thickness as the droplet. The 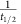 increases by a factor of ∼10 for a 0.5 μm compared with a 13 μm droplet, which implies a substantial increase in the rate of sub-micron-size droplets relative to bulk. The dashed red line in Fig. 4a simulates the behaviour of a hypothetical droplet excluding the influence of nanofocusing and light scattering but still accounting for the droplet-size-dependent absorption (see Methods, Calculation of droplet photokinetics). This curve represents bulk behaviour. The comparison with the full blue line clearly shows the pronounced influence of light focusing on the rate in the sub-micron to micron size range.

The statistically evaluated experimental first half-lives (black circles in Fig. 4a) are determined from time-dependent PA measurements (see Methods, Statistical analysis). The experimental results clearly follow the size-trend predicted by the model (full blue line). The pronounced maximum of 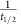 at a droplet size of ∼0.5 μm is clearly visible even though the data scatter notably below ∼1 μm (Supplementary Fig. 6) mainly because of the uncertainty in the droplet size determination (Methods, Droplet Sizing). Our experimental data show somewhat higher values of the inverse half-life for larger droplets than the model prediction. Deviations from the model assumptions including modified PA response in large droplets^47^ could potentially account for this. We have recently introduced a broad-band scattering method for accurate sizing of submicrometre particles, which will allow us in future to significantly reduce the size uncertainty in the submicrometre range (unpublished data). However, already at the current level of accuracy, the data in Fig. 4a provide the first direct observation of the strong influence of nanofocusing of light on the photokinetics in droplets.

## Discussion

The experimental results in Fig. 4 confirm a strong size-dependence of the rate of photochemical reactions in droplets. This optical phenomenon shows the most pronounced effect in the submicrometre to micrometre droplet size range for electromagnetic radiation in the UV to VIS range, that is, for the relevant frequency range in atmospheric processes. Classical cavity electrodynamics provides a semi-quantitative description of the kinetics for our ideal model system. The photokinetics of our model system is representative of typical atmospheric aerosols; that is, of typical optical properties of these particles. For example, similar quantitative results are predicted for aqueous droplets (Supplementary Fig. 7). The acceleration of the kinetics we find in the visible range is predicted to be even more pronounced in the UV range of the solar spectrum (Supplementary Fig. 7). Many aerosol particles are non-spherical. However, for particles with different shapes but the same volumes one finds quantitatively similar nanofocusing effects as for droplets. Nanofocusing also affects surface reactions since the strong intensity enhancement in forward direction shown for the internal field in Fig. 4b extends to the external field near the surface (not shown). The ability to measure and thus quantify the kinetics of the light-induced step in photochemical reactions in aerosol particles is of fundamental importance for atmospheric chemistry, where chemical processes are largely driven by sunlight. The diverse and complex processes (for example, transport and surface phenomena) in atmospheric aerosol particles require direct measurement methods as the one introduced here because simple models are of limited applicability.

The introduction of single-droplet PA spectroscopy in the present study finally makes the direct observation of the photoactive step possible. Single-droplet PA was previously deemed not feasible because of sensitivity and background issues. Here we demonstrate the viability of this new method and its very high sensitivity (*C*_abs,min_=0.0037 μm^2^) enabling studies even of single nanodroplets (10 al). PA spectroscopy provides a general absorption method that can be used in any frequency range. The combination with laser trapping lets us follow the evolution of individual droplets under controlled conditions over extended periods of time (up to several days). This versatility enables fundamental studies on many different droplet systems relevant to atmospheric and technical processes. The investigation of droplet photokinetics is just one example where this new broadly applicable single-droplet method can make an important contribution.

## Methods

### PA measurements with microphone

The PA cell is made of brass and consists of a longitudinal PA-resonator (length 40 mm, diameter 4 mm), which is connected to two buffer volumes with acoustical baffles for sound insulation (Fig. 1a)^45^. A sensitive microphone (EK 23029, Knowles) is used with a custom-made preamplifier. The output signal is recorded by a lock-in amplifier (Stanford, SR 830).

### PA measurements with tuning fork

The distance between the two tines of the tuning fork (Q 32.768 kHz TC 38, AURIS) is 300 μm. Each tine has a width of 600 μm, a thickness of 340 μm and a length of 3.8 mm. The quartz tuning fork acts as the resonant acoustic transducer, which generates an electric signal on resonant excitation by an acoustic wave due to the piezoelectric effect^46^. The signal recording is identical to the microphone set-up except for the more precise reference frequency adapted to the higher *Q*-factor.

### Aerosol generation and materials

To study photokinetics in single droplets, solutions of the photoactive dye VIS441 (Cyanine dye with formula NaC_17_H_25_N_3_O_5_S_3_ and molar mass 470, QCR solutions) in TEG solvent (ACROS organics, 99.5%) are nebulized with a medical nebulizer (Pari, PARI Boy SX). A concentration of 4.55 gl^−1^ VIS441 in TEG is used. For measurements on pure solvent droplets, pure TEG is nebulized. The Supplementary Fig. 4 shows an UV/VIS spectrum of a bulk solution of VIS441 in TEG and of pure TEG solvent, respectively.

### Droplet sizing

The particle size is determined from excitation laser light scattered elastically by the droplet. The scattered light intensity is collected for scattering angles between 76.5 ˚ and 103.5 ˚ and focused onto a CMOS camera (Thorlabs, DCC1645C, 1280 × 1024 pixels) using a camera objective (Super Carenar, focal length=50 mm, *f*-number=1.7). The particle size is retrieved by fitting calculated phase functions to experimental ones using Mie theory^41^,^44^. The sizing of sub-micron-sized droplets is difficult because only few fringes are left in the scattering pattern (for example, Fig. 1e). Larger particles exhibit brighter scattering images and many more fringes (Supplementary Fig. 3), which makes sizing easier. We estimate uncertainties in the droplet radius of about half the wavelength.

### Calculation of droplet absorbance

The PA signal *S* is assumed to be proportional to the power *P*_abs_ absorbed by the droplet, which is located at the centre of a Gaussian excitation laser beam (beam waist radius of 87 μm and cross-section *q*_L_=11,889 μm^2^) with incident power *P*


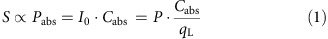


*I*_0_ is the intensity incident on the droplet and *C*_abs_ is the droplet's absorption cross-section. The equivalent absorbance *A* due to absorption is given by


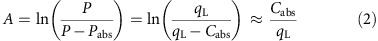


For a single droplet in the PA cell, the equivalent absorption coefficient is given by,


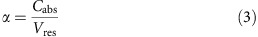


where *V*_res_=0.5 cm^3^ is the volume of the PA-resonator. The absorption cross-section of the droplet is calculated from the Mie theory^44^ with a refractive index of the surroundings equal to 1:


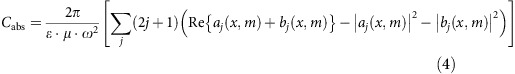


Here *a*_*n*_ and *b*_*n*_ are the scattering coefficients, 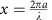 is the size parameter, *a* is the droplet radius, *λ* its wavelength of light in vacuum, *ω* is the angular frequency of the light, *ɛ* and *μ* are the permittivity and the permeability, respectively, of the droplet, and *m*=*n+ik* is the droplet's complex index of refraction at the wavelengths of the excitation laser (*λ*=445 nm). The latter is determined from UV/VIS absorption and refractometric measurements of VIS441/TEG bulk solutions and a pure TEG solution using Kramers–Kronig inversion. The refractive index of the VIS441/TEG solution for a dye concentration 4.55 gl^−1^ and the pure TEG solvent are *n+ik*=1.463+*i*·0.0062 and *n*_s_*+ik*_s_=1.460+*i·*0.0000, respectively. The refractive index of the VIS441/TEG solution (dye concentration 4.55 g l^−1^) in the UV/VIS range is provided in the Supplementary Fig. 5. For other dye concentrations (see photokinetics), it is assumed to depend linearly on the dye concentration.

### Calculation of droplet photokinetics

The droplet photokinetics is described by the following rate equation


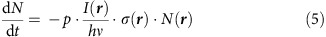


with the number density of reactant molecules *N*, Planck's constant *h*, excitation laser frequency *ν*, molecular absorption cross-section *σ* and the quantum yield *p*. Here ***r*** denotes the location within the droplet and *I* is the local field intensity. Both *I* and *σ* depend on the complex index of refraction, which in turn depends on the number density *N*, so that the rate law is no longer pseudo first order. The power absorbed by the droplet is given by the rate of absorption integrated over the droplet's volume *V*


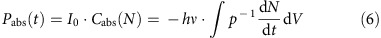


Assuming fast diffusion, that is, 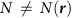, we obtain:


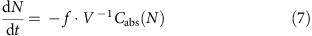


where 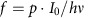 is the product of incident photon flux and reaction probability. Equation (7) is integrated using a 4th order Runge–Kutta method with the time-dependent PA signal given by equation (1). The corresponding inverse first half-lives of the PA signal as a function of droplet radius are shown as a full blue line in Fig. 4a.

To illustrate the effect of nanofocusing, we compare the above model (full blue line in Fig. 4a) with a model that neglects the influence of nanofocusing (dashed red line in Fig. 4a). This model is obtained from equations (5) and (6)


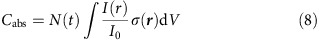


by inserting the small particle limit^44^ for *σ*


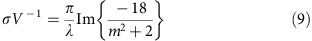


and a Beer–Lambert expression for the intensity distribution within the particle


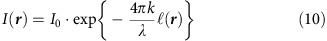


where 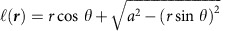 is the absorption path length at distance *r* from the centre of the particle and at polar angle *θ* relative to the incident beam direction.

### Statistical analysis of experimental photolysis data

To account for the uncertainties both in the particle radii and in the decay half-lives of the experimental data set (Supplementary Fig. 6), we perform a two-step maximum likelihood analysis. First, the distribution of particle radii 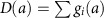 is analysed assuming a normally distributed error for the size determination,


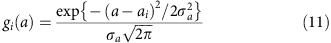


with a constant s.d. of *σ*_*a*_=220 nm. The local extrema in *D* at *a*_*k*_ divide the size range into sections with a lower and an upper half for each cluster of data, which are combined into a single section for isolated data points. For each section, we finally obtain the most probable values for particle radius and the inverse half-life as weighted averages over the particles with weights given by,


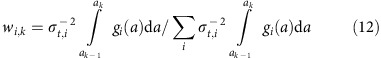


This implies normally distributed errors for the experimental inverse half-lives with s.d. *σ*_*t,i*_ ranging from about 10% for the most accurate measurements to about 50% for measurements with 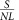
<<10 (typically small particles). The error bars in Fig. 4a were obtained by s.e. propagation.

## Additional information

**How to cite this article:** Cremer, J. W. *et al*. Photoacoustics of single laser-trapped nanodroplets for the direct observation of nanofocusing in aerosol photokinetics. *Nat. Commun.* 7:10941 doi: 10.1038/ncomms10941 (2016).

## Supplementary Material

Supplementary Information Supplementary Figures 1-7
