# Supplementary material for: Photoacoustics of single laser-trapped nanodroplets for the direct observation of nanofocusing in aerosol photokinetics
Source: Nat Commun. 2016 Mar 16;7:10941. doi: 10.1038/ncomms10941 (PMC4799369; doi:10.1038/ncomms10941)
Supplement: Supplementary Information — Supplementary Figures 1-7 [file ncomms10941-s1.pdf]

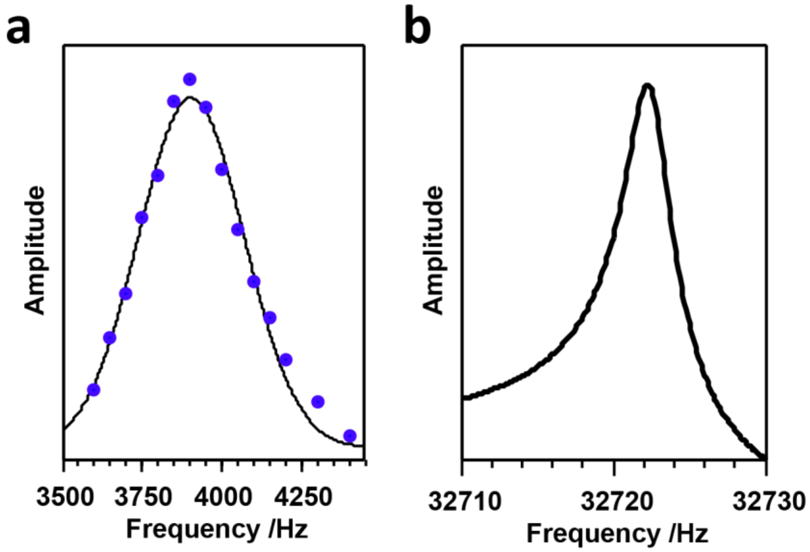

Supplementary Figure 1| Acoustic response as a function of the modulation frequency. (a) For the PA cell. Blue dots: experimental values. Black line: Gaussian fit. **(b)** Experimental data for the quartz tuning fork. The data are recorded at a temperature of 293 K.

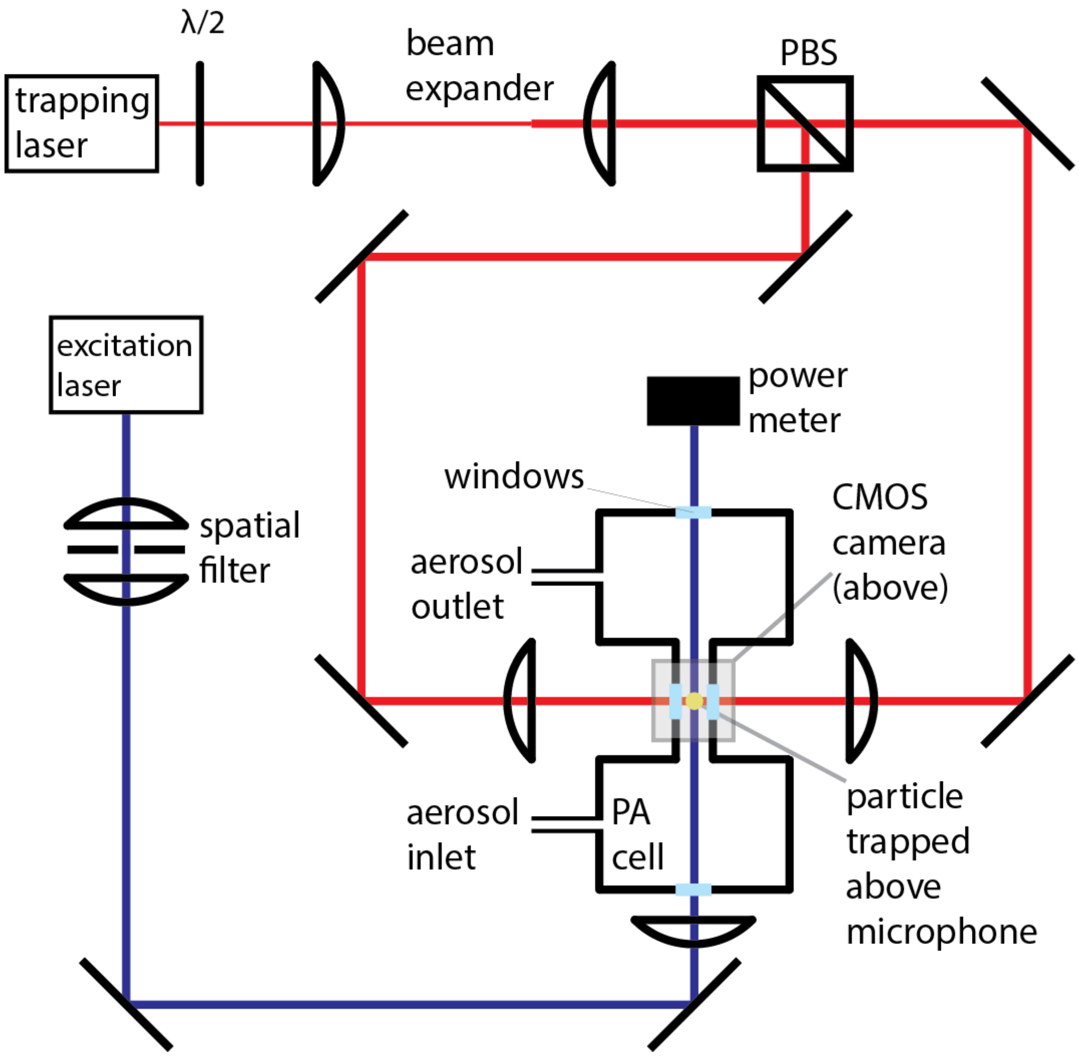

Supplementary Figure 2| Scheme of the overall experimental setup for the microphone setup. The scheme shows the resonant PA cell with microphone, windows for laser passage, aerosol in and outlet. A CMOS camera is used for droplet imaging and sizing (Fig. 1e and Supplementary Fig. 3). The excitation laser (λ = 445 nm; < 40 mW) is focussed with a lens to a 87 µm radius beam size (85% Gaussian intensity) at the location of the droplet. The counter-propagating optical tweezer is built from a continuous laser beam (λ = 660 nm; 200 mW) split by a beam splitter cube (PBC) into two cross-polarized arms, which are focused into the centre of the trap by two aspheric lenses with effective numerical apertures of 0.1.

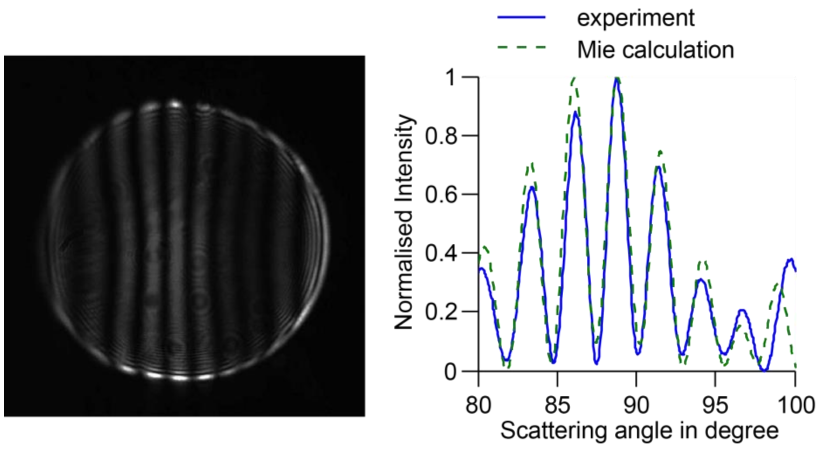

Supplementary Figure 3| Light scattering of a 1.9 µm VIS441/TEG solution droplet. Light scattering image as recorded by the CMOS camera (left) with experimental and calculated phase function (right). Many more fringes are visible in the scattering patterns compared with the submicron-sized droplet in Fig. 1e.

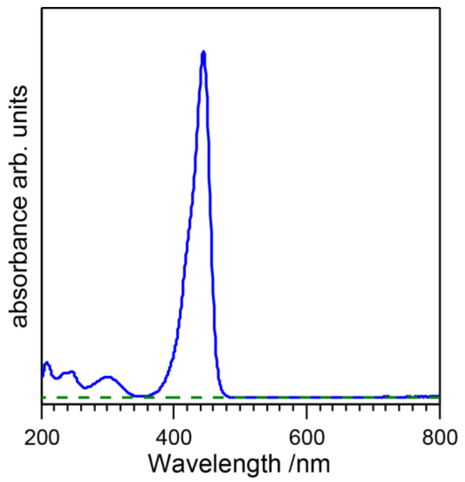

Supplementary Figure 4| UV/VIS absorption spectra. Blue full line: VIS441/TEG solution. Green dashed line: pure TEG solvent. The solution strongly absorbs at the wavelengths of the excitation laser (445 nm), while the solvent does not show any appreciable absorption.

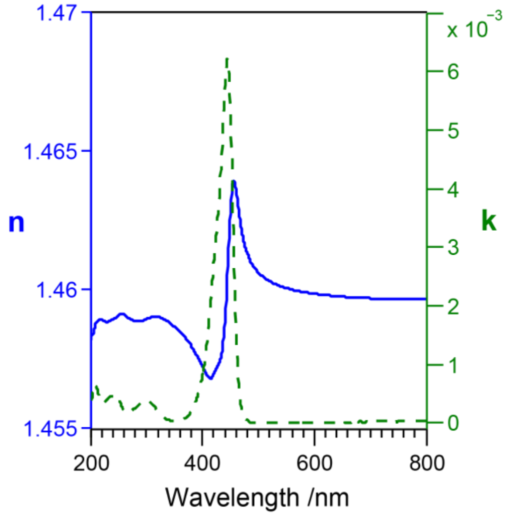

Supplementary Figure 5| Refractive index of a 4.55 gL^{-1} VIS441/TEG solution in the UV/VIS range. Full blue line: Real part *n*. Dashed green line; Imaginary part *k*. The data are determined from UV/VIS absorption (Supplementary Fig. 4) and refractometric measurements of VIS441/TEG bulk solutions using Kramers-Kronig inversion [Hawranek *et al.*, Spectrochim. Acta A, 32, 85, (1976)]. The solution does not absorb at the trapping wavelength of 660 nm.

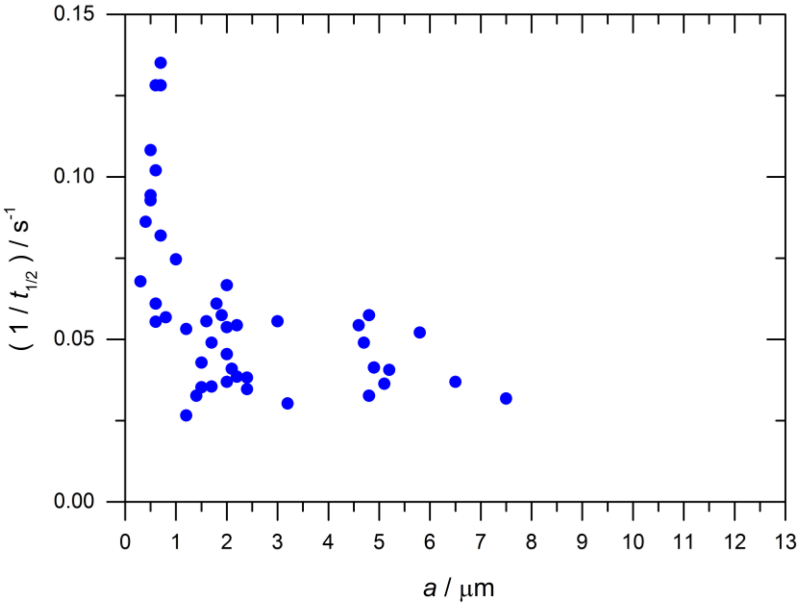

Supplementary Figure 6| Experimental raw data for first half-lives for 1 mW laser power. The raw data are determined from PA measurements recorded during the photolysis of the

absorber (see examples in Fig. 3). The smallest and the largest droplet radii are about 300 nm and 7.5 µm, respectively. We estimate uncertainties in the droplet radius of about half the wavelength of the laser used for the scattering experiments (Methods, droplet sizing). The statistically evaluated data in Fig. 4a are obtained from the raw data as described in Methods, Statistical analysis of experimental photolysis data.

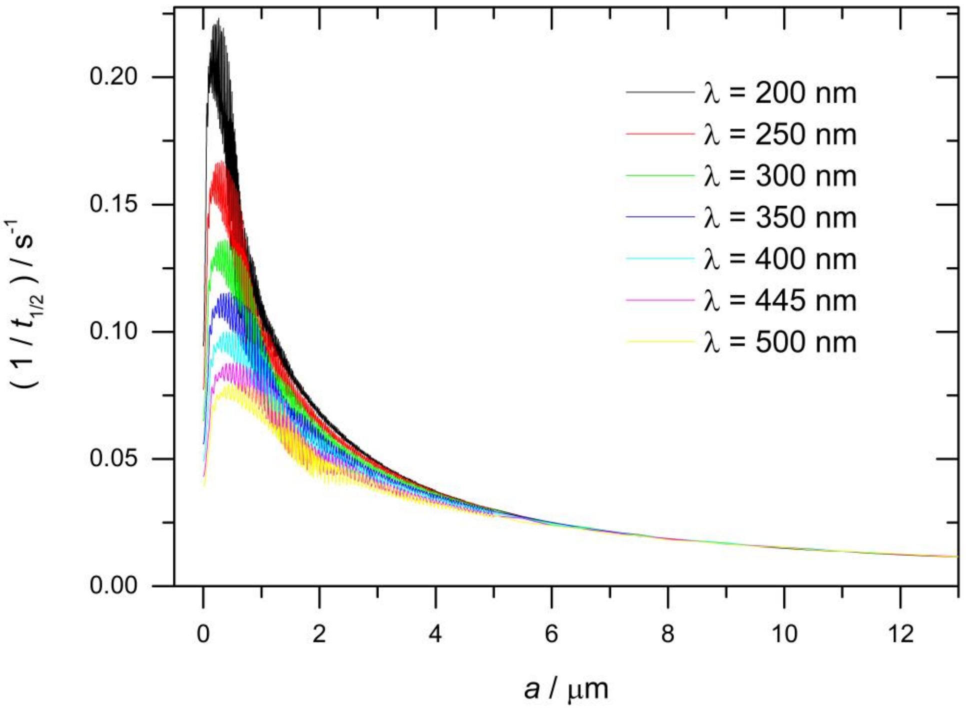

Supplementary Figure 7| Model calculations for photokinetics in aqueous droplets. Inverse half-lives for aqueous droplets as a function of the droplet radius *a*. The pink line for λ = 445 nm shows that the effect in water is quantitatively similar to TEG solvent (Fig. 4a). The calculations for different wavelengths λ show that the acceleration in the rate is even more pronounced in the ultraviolet than in the visible. The calculations are based on wavelength-dependent real refractive indices of water, which vary between n = 1.396 at λ = 200 nm and n = 1.335 at λ = 500 nm. All data are for an initial imaginary refractive index of k = 0.0062.
